# Diet gel-based oral drug delivery system for controlled dosing of small molecules for microglia depletion and inducible Cre recombination in mice

**DOI:** 10.1038/s41684-025-01617-1

**Published:** 2025-09-26

**Authors:** Joel Jovanovic, Megan L. Stone, Samantha R. Dooyema, Yuankai K. Tao, Sabine Fuhrmann, Edward M. Levine

**Affiliations:** 1 Vanderbilt Eye Institute, Vanderbilt University Medical Center, Nashville, TN USA; 2 Department of Ophthalmology and Visual Sciences, Vanderbilt University, Nashville, TN USA; 3 Department of Cell and Developmental Biology, Vanderbilt University, Nashville, TN USA; 4 Department of Biomedical Engineering, Vanderbilt University, Nashville, TN USA

**Keywords:** Genetic models, Animal biotechnology, Drug delivery

## Abstract

Small molecules such as PLX5622 for microglia depletion and tamoxifen for inducible Cre recombination are commonly used in mouse research. Traditional application methods such as drug-infused chow, oral gavage or injections have limitations, including uncontrolled dosing (chow) or risk of injury and/or stress (gavage or injections). Here, to address these issues, we have developed an alternative oral drug delivery system using a gel-based rodent maintenance diet that allows for controlled consumption and adjustment of dosage and is suitable for water-insoluble small molecules. We tested DietGel 93M (93M) infused with PLX5622 (0.8 mg/g and 2.0 mg/g) in the *Cx3cr1*^*gfp/+*^ retinal microglia reporter mouse and tamoxifen-infused 93M (0.3125 mg/g) in the Rlbp1-CreERT2*;Rosa*^*ai14*^ mouse with an inducible tdTomato reporter in retinal Müller glia. Mice were single caged and received daily batches of PLX5622-infused 93M over 14 days or tamoxifen-infused 93M for 1 or 3 days followed by a 14-day observation period. Longitudinal scanning laser ophthalmoscopy in vivo and fixed-tissue imaging were used to track GFP and tdTomato expression. Following evaluation of a suitable 93M consumption rate to sustain body weight, the PLX5622-93M diet at both concentrations tested showed a 94% microglia depletion rate at 3 days and >99% after 1 and 2 weeks. The tamoxifen-93M diet confirmed suitability for inducible Cre recombination, with significant treatment time-dependent efficacy and a positive correlation between total tamoxifen dose and tdTomato expression. This study demonstrates that a diet gel-based drug delivery system offers a minimally invasive alternative to current drug application methods for PLX5622 and tamoxifen. This approach could be useful for other drugs or tissues beyond the retina.

## Abstract (web summary)

Jovanovic et al. show that a gel-based diet can be used as a minimally invasive alternative system for the delivery of small molecules for microglia depletion and inducible Cre recombination in mice.

## Main

Over the past years, different drug delivery systems for rodents have been established in biomedical research. The preferred route of delivery often depends on the drug’s properties, drug vehicle, required dose, research question and rodent condition^1,2^. Among the most common systemic delivery methods that use the oral route are gavage, drinking water or hydration gels and drug-infused chow. Other drugs require bypassing the first-pass effect by using the parenteral route, such as intravenous (tail vein), subcutaneous or intraperitoneal (i.p.) injections^1,3–6^. Even though these systems are widely used, they have disadvantages that must be considered such as the risk of injury, stress to the animal and control over dosage. Although methods such as micropipette-guided drug administration and automated systems such as PiDose have been developed to mitigate these risks, they still present additional challenges, such as the installation of feeding devices or direct interactions with animals^7,8^.

Recently, a broad range of small molecules became available for a variety of purposes^9,10^. PLX5622 for microglia depletion and tamoxifen for CreER recombination are commonly used small molecules in biological research^11–16^. PLX5622 is a small-molecule kinase inhibitor that selectively blocks the colony-stimulating factor 1 receptor (CSF1R) signaling required for cell survival in monocytes and in microglia and macrophages in the central nervous system^17^. Tamoxifen is a selective estrogen receptor modulator widely used in combination with a Cre recombinase−LoxP system for tissue-specific and temporal gene regulation in mice^18–20^.

Owing to the water-insoluble properties of both drugs, the standard oral application methods for these small molecules include drug-infused chow (ad libitum) for PLX5622 and tamoxifen or oral gavage for tamoxifen. However, these methods may not always be suitable. While chow lacks control over dosage (mg/g) and consumption rate (g/day) between rodents, oral gavage risks injury and stress to the animals. Therefore, an alternative that mitigates these effects is needed. In this study, we have developed a diet gel-based oral drug delivery system that allows for dosage adjustment and is suitable for water-insoluble small molecules using the complete gel-based maintenance diet ClearH_2_O DietGel 93M (93M).

## Results

### A 93M DietGel consumption rate of 8 g/day allows for complete consumption and steady body weight

To facilitate the transition from regular chow to diet gel and to allow for a time-matched start of the experiment across all conditions, mice were fasted for 16 h before the experimental start. Body weight was tracked throughout the experiments with the post-fast body weight serving as the baseline. This allowed us to directly assess the effect of the diet gel with and without drug infusion.

**Fig. 1:**
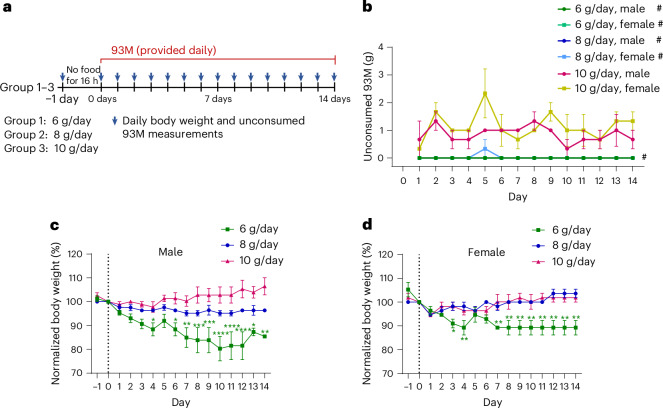
Consumption rate determination for steady body weight and complete consumption of provided 93M in B6129SF1/J mice. **a**, Experimental design of feeding groups receiving either 6, 8 or 10 g of drug-free 93M every 24 h over 14 days. *n* = 6 mice (3 males, 3 females) per feeding group. Unconsumed 93M (g) and body weight (g) were measured after each 24 h feeding cycle, including a body weight measurement before (day −1) and after (day 0) an initial fasting period of 16 h. **b**, Unconsumed 93M (g) after each feeding cycle per feeding group and sex (*n* = 3). The # symbol indicates data points at 0 g of unconsumed 93M. **c**,**d**, Temporal body weight measurements normalized and compared with the post-fasting body weight (day 0) in males (**c**) and females (**d**) (*n* = 3). Two-way ANOVA with Dunnett’s multiple comparisons tests were performed for **c** and **d**. The results are shown as mean ± s.e.m (**b**,**c**,**d**). Significance levels: *P < 0.05, **P < 0.01, ***P < 0.001 and ****P < 0.0001.

As a first step, we determined the optimal daily consumption rate of drug-free 93M by assigning three feeding groups to receive either 6, 8 or 10 g/day of 93M over 14 days (Fig. 1a). This experiment used B6129SF1/J mice, which share the genetic background with the two strains used for the following drug-incorporation studies. Body weight and the residual amount of food (unconsumed 93M) were measured daily after each feeding cycle, including a body weight measurement right before and after an initial fasting period of 16 h before the 93M feeding start. Each feeding group included three male and three female mice, and all mice were single caged for the experiments.

Figure 1b shows the mean amount of unconsumed food at each time point for males and females. For both sexes, 6 and 8 g were completely consumed over the course of the experiment, except for one female on day 5. By contrast, unconsumed food was left behind every day with 10 g/day. This shows that males and females consistently consume 6 or 8 g/day, and leave food behind when given 10 g/day, with an average of 0.86 ± 0.09 g/day for males and 1.14 ± 0.12 g/day for females without a significant difference between them (*P* = 0.0588, two-tailed unpaired *t*-test).

A potential confound that could underestimate the amount of unconsumed food and lead to the miscalculation of an ingested drug dose is evaporation. Our temporal weight measuring data of 93M at 6, 8 and 10 g held in ventilated cages show mass reductions between 25% and 40% over 24 h, depending on the initial amount of 93M and on the drug–vehicle mix-in (Supplementary Fig. 1). However, we observed that mice in the 6 g/day and 8 g/day groups did not leave food residues in the Petri dishes, indicating that they consumed the full portions. For the 10 g/day group, however, the true mass of the unconsumed 93M could be greater than what was measured due to the uncertainty of when the food was consumed over a 24-h period.

To determine the effect of the three different provided amounts of 93M on body weight maintenance, animals were weighed daily, and the measurements were normalized to the initial post-fasting weights for each animal (day 0) (Fig. 1c,d). Two-way analysis of variance (ANOVA) shows that both sexes had significant variations in body weight changes depending on the feeding amount (*P*_males_ < 0.0001, *P*_females_ < 0.0001) and time (*P*_males_ = 0.0036, *P*_females_ = 0.0013). These significant changes were specifically observed in mice given 6 g/day, which caused significant drops in body weights, with one male mouse removed from the study on day 12 due to a weight loss greater than 30%.

Overall, both males and females fed 8 g/day of 93M showed a steady body weight over time and completely consumed the daily portions. Therefore, we decided to use 8 g/day of 93M for the following drug incorporation studies.

### Efficient retinal microglia depletion kinetics in mice treated with low and high-dose PLX5622-infused 93M DietGel

**Fig. 2:**
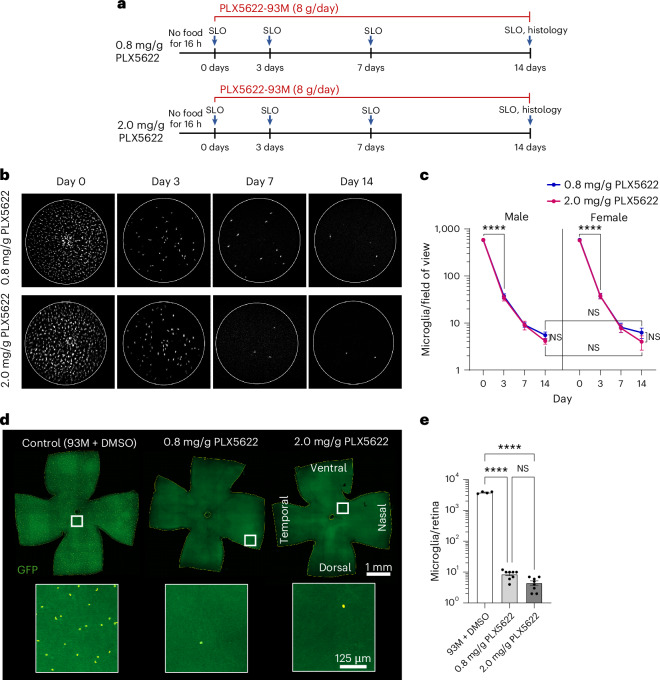
Feasibility and efficacy evaluation of PLX5622-infused 93M for retinal microglia depletion in GFP-expressing microglia reporter mice. **a**, Experimental design with two PLX5622-93M dosage groups (0.8 mg/g and 2.0 mg/g) in heterozygous B6.129P2(Cg)-*Cx3cr1*^*tm1Litt*^/J mice receiving 8 g/day over 14 days, including daily body weight and unconsumed food measurements. SLO was performed on days 0, 3, 7 and 14. *n* = 8 mice (4 males, 4 females) per PLX5622 dosage group. **b**, Representative longitudinal SLO image series per treatment group shows retinal microglia depletion in vivo. **c**, Quantification of microglia counts in SLO images per sex and dosage group (*n* = 8 eyes per group and sex). **d**, Representative endpoint histology (day 14) of whole-mounted retina per dosage group, including a control retina treated with DMSO-infused 93M. The inset shows a magnified section with masked microglia (yellow boundary) for better visualization. **e**, Quantification of microglia counts in retinal whole mounts over both sexes per dosage group (*n* = 8), including a DMSO-infused control group (*n* = 4). Two-way ANOVA with Tukey’s multiple comparisons test were performed for **c** and one-way ANOVA with Tukey’s multiple comparisons test for **e**. The results are shown as mean ± s.e.m. ****P < 0.0001. NS, not significant.

The feasibility and efficacy of PLX5622-infused 93M (PLX5622-93M) treatment were tested in *Cx3cr1*^*gfp/+*^ microglia reporter mice split into a low (0.8 mg/g) and a high (2.0 mg/g) dosage group with feedings of 8 g/day over 14 days (Fig. 2a). Body weights and unconsumed food were measured daily. Both groups included eight mice with four males and four females. Scanning laser ophthalmoscopy (SLO) was used to visualize the microglia depletion kinetics in vivo at days 3, 7 and 14 compared with day 0 (pretreatment baseline). Endpoint histology in retinal whole mounts was used to confirm the in vivo results.

SLO proved the feasibility of 93M as a drug delivery system for PLX5622, as evidenced by the loss of GFP^+^ cells, the readout for microglia depletion (Fig. 2b). Surprisingly, both dosages showed significant depletion after 3 days of treatment (*P* < 0.0001, two-way ANOVA with Tukey’s multiple comparison test) with similar depletion kinetics for both sexes (Fig. 2c). Animals treated with 0.8 mg/g showed a 93.6% reduction in microglia at day 3, 98.5% at day 7 and 99.1% at day 14. Animals treated with 2.0 mg/g showed a 93.8% reduction in microglia at day 3, 98.6% at day 7 and 99.3% at day 14.

Quantification of fixed retinal whole-mount tissues on day 14 confirmed these findings (Fig. 2d,e), with a depletion of 99.8% for the low dose and 99.9% for the high dose when compared with the vehicle-treated control group. In alignment with the SLO findings on day 14, there was no significant difference between both dosages over both sexes, with an adjusted *P* value of 0.9973, while both dosages had a significant effect in comparison to the DMSO-93M control group (*P* < 0.0001, one-way ANOVA with Tukey’s multiple comparisons test) on day 14.

Consumption of the PLX5622-93M was complete throughout the experiment, except for the first two feeding cycles in females (Supplementary Fig. 2a). Interestingly, the females that received either dosage and the males treated with the low dose regained their initial pre-fasting body weight over the treatment course, while the males treated with 2.0 mg/g stayed at the level of the post-fasting weight (Supplementary Fig. 2b,c). Consistent with this, the males treated with the higher dose had significantly lower normalized body weights from day 4 onward compared with the other groups (Supplementary Fig. 2d). These observations reveal a specific dose-dependent effect of PLX5622 in male mice that extends beyond microglia depletion (Discussion).

To exclude potential effects on the microglia counts caused by the drug vehicle DMSO in the 93M, a separate SLO imaging session was conducted with 93M infused with DMSO only (Supplementary Fig. 2e). The mice received 8 g daily feedings of DMSO-infused 93M and were imaged at day 0 (pretreatment) and on days 7 and 14 of the treatment. The microglia counts in the in vivo images showed, in comparison to the pretreatment condition (578.50 ± 11.49), no effect on the total number of microglia present on day 7 (573.25 ± 19.77; *P* = 0.9963, one-way ANOVA with Dunnett’s multiple comparisons test) and day 14 (586.75 ± 13.92; *P* = 0.9154, one-way ANOVA with Dunnett’s multiple comparisons test).

### Tamoxifen-infused 93M DietGel-treated mice successfully induce tdTomato expression in retinal Müller glia

**Fig. 3:**
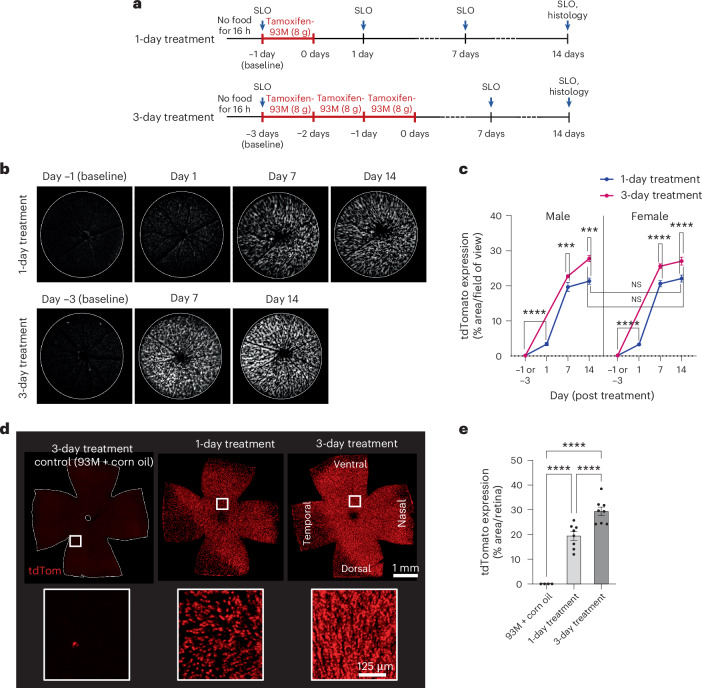
Feasibility and efficacy evaluation of tamoxifen-infused 93M for Cre recombination using a mouse line with inducible tdTomato expression in retinal Müller glia. **a**, Experimental design for 1-day and 3-day tamoxifen-93M treatment groups in Rlbp1-CreERT2;*Rosa*^*ai14*^ mice. Both groups received 8 g/day with an equal tamoxifen concentration of 312.5 µg/g. The body weight and unconsumed tamoxifen-93M were measured after each feeding cycle. SLO was performed for the 1-day treatment on days −1 (baseline), 1, 7 and 14, and on days −3 (baseline), 7 and 14 for the 3-day treatment. *n* = 8 mice (4 males, 4 females) per tamoxifen treatment group. **b**, Representative longitudinal SLO image series per treatment group shows tdTomato expression induction in vivo. **c**, Quantification of the TdTomato expression as an area fraction of positive pixels in SLO images per sex and treatment group (*n* = 8 eyes per group and sex). **d**, Representative endpoint histology (day 14) of whole-mounted retina per treatment group, including a control retina treated with corn oil-infused 93M. The reduced tdTomato expression in the dorso-temporal area is a feature of the Rlbp1-CreERT2;*Rosa*^*ai14*^ line that is independent of tamoxifen administration route (unpublished data). **e**, Quantification of tdTomato expression as an area fraction of positive pixels in retinal whole mounts over both sexes per treatment group (*n* = 8), and a 93M + corn oil control group (*n* = 4). Two-way ANOVA with Tukey’s multiple comparisons test was performed for **c** and one-way ANOVA with Tukey’s multiple comparisons test for **e**. The results are shown as mean ± s.e.m. ***P < 0.001, ****P < 0.0001.

To test for feasibility and control over the level of Cre recombination using tamoxifen-infused 93M (tamoxifen-93M), we used Rlbp1-CreERT2*;Rosa*^*ai14*^ mice, which express tdTomato in Müller glia after tamoxifen treatment^21–23^. Two treatment groups were designed to test for different tamoxifen exposure times: one with a short (1-day treatment) and one with a long exposure (3-day treatment) (Fig. 3a). All mice received 8 g of tamoxifen-infused 93M per day of treatment with a tamoxifen concentration in the food of 312.5 µg/g. This daily dose is approximately equivalent to a single dose of 100 µg/g body weight by oral gavage in a mouse that weighs 25 g. Each group included eight mice with four males and four females. Longitudinal SLO imaging was performed to detect Cre-dependent tdTomato expression induction in retinal Müller glia. Baseline expression levels were established before treatment began. Both groups were followed up 7 and 14 days post-tamoxifen treatment, including a 1 day post-treatment time point for the 1-day treatment group. Endpoint histology was used to confirm the tdTomato expression levels in fixed retinal whole mounts on day 14.

SLO confirmed successful tamoxifen delivery and Cre recombination, as shown by the induction of tdTomato expression over time (Fig. 3b). Interestingly, the 1-day treatment showed a significant increase (*P* < 0.0001, two-way ANOVA with Tukey’s multiple comparisons test) in the tdTomato expression for males and females after 24 h compared with baseline, with a relative expression increase from 0.12 ± 0.04% to 3.39 ± 0.45%, and 0.14 ± 0.03% to 3.29 ± 0.21%, respectively (Fig. 3c). The tdTomato expression continued to increase until the end of the experiment on day 14 to 21.35 ± 0.88% for the males, and to 22.09 ± 0.98% for the females, without a significant difference between both sexes. The 3-day treatment caused a small but significant increase in tdTomato expression on day 14 compared with the 1-day treatment, with a relative value of 27.78 ± 0.86% for the males (*P* = 0.007, two-way ANOVA with Tukey’s multiple comparisons test) and 27.05 ± 1.12% for the females (*P* < 0.0001) (Fig. 3c). No significant difference was observed on day 14 between sexes within the same treatment regimes.

The endpoint evaluation of the tdTomato expression level in the whole-mounted retina supported the treatment time-dependent effect shown by the in vivo findings (Fig. 3d,e). The calculated overall tdTomato expression for males and females following the 1-day and 3-day treatment was 19.38 ± 1.72% and 29.31 ± 1.76%, respectively, with a significant difference between treatment times (*P* = 0.0010, one-way ANOVA with Tukey’s multiple comparisons test).

To control for potential effects caused by the corn oil (drug vehicle) in 93M, a separate SLO study was conducted using the 3-day treatment paradigm with corn oil-infused 93M (Supplementary Fig. 3a). The mice were imaged pretreatment (baseline) and at days 7 and 14 post-treatment. The evaluation of the tdTomato expression in vivo showed no significant differences at both time points when compared with the baseline conditions, with a relative expression of 0.13 ± 0.04% at baseline and 0.09 ± 0.03% and 0.10 ± 0.01% on days 7 and 14, respectively. In comparison, the endpoint evaluation of the whole-mounted retina showed an average tdTomato expression of 0.02 ± 0.01% (Fig. 3e).

**Fig. 4:**
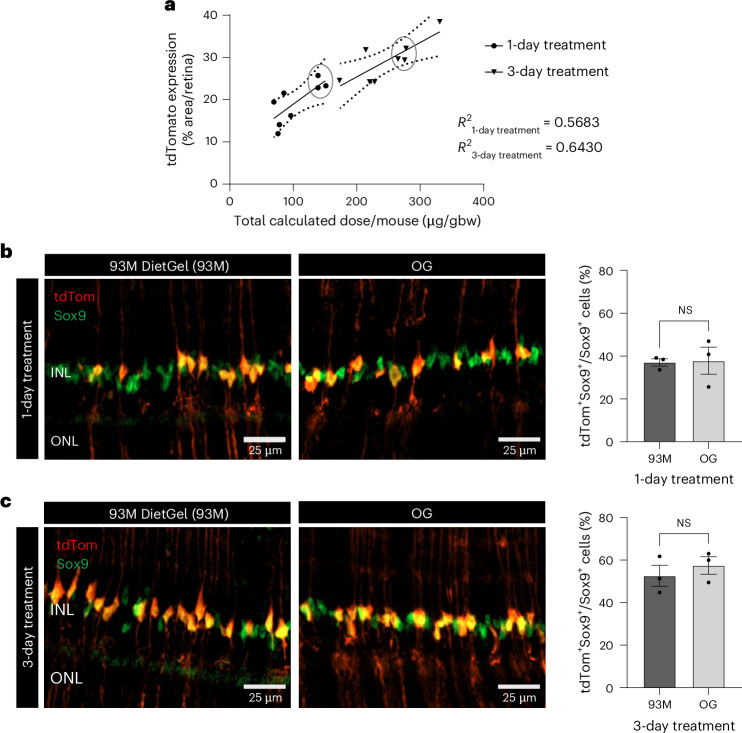
Dose–response curve of tamoxifen-infused 93M and efficacy evaluation. **a**, Linear regression analysis of the calculated total dose of tamoxifen for each animal (µg/gbw) versus the tdTomato expression level present in the retinal whole mounts of the 1-day and 3-day treatment groups on day 14 (*n* = 16). The total tamoxifen dose per mouse (µg/gbw) was calculated based on the total amount (g) of consumed tamoxifen-93M and the averaged body weights over the treatment time (1 or 3 days). The *R*^2^ for both treatment groups and 95% confidence intervals are shown. **b**,**c**, Tamoxifen-induced Cre recombination efficacy comparison between the 93M DietGel (93M) and the OG drug delivery system. For each 93M treatment regime (1-day and 3-day), three animals with similar tamoxifen doses were identified in **a** (circled dots or triangles, respectively), with an average dose of 143 µg/gbw for the 1-day treatment (**b**) and an average cumulated dose of 273 µg/gbw for the 3-day treatment (**c**), respectively. The doses for the OG comparison groups were matched (*n* = 3 mice per group), with the 3-day treatment group receiving three gavages with 91 µg/gbw, each given 24 h apart. The efficacies for the 1-day (**b**) and the 3-day (**c**) treatment groups were calculated by the percentage of nuclear Müller glia marker Sox9^+^ and tdTomato^+^ cells over all Sox9^+^ cells in retinal sections of the right eye (*n* = 3 mice per condition). A two-tailed Mann−Whitney *U* test was performed for **b** and **c**. The results are shown as mean ± s.e.m. INL, inner nuclear layer; ONL, outer nuclear layer of the retina.

Unlike for the PLX5622-93M feeding, tamoxifen in 93M caused variations in the consumption rate between animals, with a tendency for females to leave more unconsumed tamoxifen-93M after each feeding cycle (Supplementary Fig. 3b), but without significant body weight changes during the short treatment periods in comparison to the post-fasting conditions (Supplementary Fig. 3c,d). Since the difference in consumption could result in differences in tamoxifen dosages between animals, a dose–response curve was calculated using a simple linear regression analysis of the total dose for each animal versus the tdTomato expression present in the retinal whole mounts on day 14 (Fig. 4a). The total tamoxifen dose per mouse (µg/g body weight) was calculated by the total amount (g) of consumed tamoxifen-93M and the averaged body weights over the treatment time (1 or 3 days). Both treatment groups show a significant correlation between the dose and the tdTomato expression with an *R*^2^ of 0.5686 for the 1-day treatment and an *R*^2^ of 0.6430 for the 3-day treatment.

Furthermore, the dose–response curve allowed for the identification of three animals per treatment regimen with a similar calculated dose per mouse (microgram per gram body weight (gbw)), highlighted in Fig. 4a. The mean doses for the groups are 143 µg/gbw in the 1-day treatment regimen and a cumulative dose of 273 µg/gbw in the 3-day treatment regimen. To compare the efficacy of the 93M-based delivery system (93M) versus an established method, these doses of tamoxifen were delivered to mice by oral gavage (OG), a method that uses the oral route that exposes the administered tamoxifen to the same absorption and first-pass metabolism. Interestingly, the results show similar efficacies between the delivery methods for each treatment regimen (Fig. 4b,c), as shown by the mean percentage of tdTomato-expressing Müller glia cells, identified by the nuclear marker Sox9 in retinal sections. More specifically, the 1-day treatment regimen showed a mean efficacy of 37.06 ± 1.73% for 93M-based delivery and 37.79 ± 6.33% for OG (*P* = 0.7000, two-tailed Mann−Whitney *U* test) (Fig. 4b), while the 3-day treatment regimen showed a mean efficacy of 52.57 ± 4.94% and 57.45 ± 4.11% (*P* = 0.7000, two-tailed Mann−Whitney *U* test), respectively (Fig. 4c).

## Discussion

Gel-based diets are often used to supplement chow for breeding purposes or in postoperative or compromised animals to prevent body weight loss^24–29^. However, gel-based maintenance diets can also be used to fully replace chow^30,31^. In this study, we identified that daily feedings of 8 g of 93M per mouse is a suitable amount that can be completely consumed and allows for steady body weight over time in adult mice of both sexes. Feeding a lower amount, namely 6 g/day, caused a steady drop in body weight, which was more pronounced in males. This may be attributed to the higher average body weight of males with a mean difference between the sexes of 32.8 ± 2.1% on post-fast day 0 (*P* < 0.0001, unpaired two-tailed *t*-test). On the other hand, the 93M consumption data of age-matched mice indicates that females in the 10 g/day group tend, even though not significantly, to leave more unconsumed food after each feeding cycle than their male counterparts. However, due to the small sample size, it remains unclear whether these effects would remain in a study using a larger sample size.

Furthermore, we show that the gel texture and the liquefication properties of 93M upon heating allow for a simple way to infuse water-insoluble drugs into 93M. Here, we tested the two small molecules, PLX5622 and tamoxifen, with good results, and it is likely that this delivery system could work for other water-insoluble drugs or test compounds.

The PLX5622-infused 93M experiment demonstrated high microglia depletion kinetics in both concentration groups, with over 98% after 1 week of treatment. These results are comparable to or better than published chow-based depletion kinetics, with over 90% depletion after 1 week^11–13,32^. However, for a direct comparison, it is important to consider both the concentration and the amount of consumption. The standard concentration of PLX5622 in chow is 1.2 mg/g with an estimated consumption of 5 g per day, which provides a similar daily dose achieved with the low-dose PLX5622-infused 93M at 0.8 mg/g with 8 g consumed daily.

Unexpectedly, our PLX5622 experiment using the high dose of 2.0 mg/g revealed a significant dose-dependent effect on the body weights in male mice. This is a new and interesting observation that may imply sex-specific metabolic changes in males upon high-dose PLX5622 treatment, especially since PLX5622 has been shown to affect glucose metabolism and adipose tissue remodeling^33,34^. This should be considered for studies in which a comparable dose is planned.

There are several advantages of using PLX5622-infused 93M for microglia depletion. This minimally invasive approach enables the use of different drug concentrations with consistent consumption rates over time in age-matched mice of both sexes. In comparison to drug incorporation into chow, a commonly used minimally invasive delivery method for PLX5622, the 93M approach offers the ability to easily and cost-effectively adjust dosage in small batches of food. These features make it practical to test PLX5622 from different vendors or between different production lots and adjust dosages accordingly. Another advantage is the lower sucrose content of the AIN-93-based diet used here, which mainly uses corn starch as a carbohydrate source, compared with the commonly used high sucrose-based AIN-76A chow for PLX5622 and other drugs^35,36^. In addition, the presented drug delivery system avoids the risks and animal welfare concerns of invasive methods of PLX5622 applications, such as i.p. injections of PLX5622-DMSO mixtures, which require daily injections^37,38^. This method may be suitable for bigger rodents, such as Sprague Dawley rats, but we observed abdominal cyst formations during a trial of repeated PLX5622-DMSO i.p. injections into mice following a published protocol^37^. Overall, the diet gel-based delivery of PLX5622 is highly efficient, mitigates adverse effects of other treatment routes and allows for long-term exposure experiments or the testing of different batches of drugs with adjusted dosages.

We also demonstrate that tamoxifen can be infused into 93M and that feeding it to adult and sex-matched mice induces Cre recombination. Here, a single tamoxifen concentration (312.5 µg/g) and a daily feeding amount (8 g/day) were used while controlling for the treatment time (1-day versus 3-day treatment). The longitudinal SLO data observing Cre-dependent tdTomato expression in retinal Müller glia shows that tamoxifen works quickly, with tdTomato activation within 24 h after one feeding cycle. Furthermore, the control over treatment time is sufficient to significantly adjust the level of Cre recombination, despite the differences in consumption between animals. Nevertheless, the small sample size and unrecorded individual consumption behavior might introduce interindividual variability, which could explain the moderate *R*^2^ values in the dose–response curve (Fig. 4a). An alternative to better control the level of Cre recombination may be adjusting the tamoxifen concentration in 93M to the individual body weights of the mice. However, our data indicate that mice show some reluctance to tamoxifen-infused 93M, which might be a limitation on the amount of tamoxifen that can be incorporated due to palatability.

Interestingly, studies using a commercially available tamoxifen-incorporated chow reported similar observations with reduced feeding and weight loss within the first days of treatment^39–41^. In our study, we only observed a limited drop in body weight over the short tamoxifen treatment periods of 1 and 3 days relative to the preceding post-fasting body weight (Supplementary Fig. 3c,d). Additionally, mice treated with 93M or corn oil-infused 93M also showed a limited initial weight loss (Fig. 1c,d and Supplementary Fig. 4a), while this was not observed in the PLX5622-93M or DMSO-infused 93M treated mice (Supplementary Figs. 2c,d and 4b). Thus, it remains unclear whether tamoxifen, corn oil, the switch to a gel-based diet, the mouse strain or a combination of these factors contributed to the weight loss. Nevertheless, certain observed weight losses might require individual mice being removed from the study due to study design or exceeding regulatory thresholds. The weight loss may be reduced by shortening fasting time or by omitting it if the infused drug does not affect palatability. Another option may be sweetening the drug-infused 93M mixture by adding sweetened milk, similar to a previously published minimally invasive delivery method for tamoxifen^42^. Overall, a high level of recombination can be achieved with a repeated feeding regimen, whereas a single feeding can be advantageous for mosaic recombination. Importantly, tamoxifen-infused 93M offers a refinement over gavage- or injection-based delivery.

Generally, the 93M delivery system mitigates the stress of daily i.p. injections or gavages, which can be a confound in research studies using rodents^43–47^. However, single housing is required for consumption and dosage monitoring, which may present a limitation. However, in contexts where precise monitoring is not essential, such as ad libitum administration of PLX5622, single housing can be avoided. In comparison to other minimally invasive drug delivery methods, such as PiDose or micropipette-guided drug administration, the 93M approach does not require the installation of a feeding device or temporarily restraining the animal for feeding purposes^7,8,42^.

In conclusion, our study shows that a gel-based diet such as 93M can be used as a minimally invasive alternative system for drug delivery with the ability to adjust for dosing. It shows the advantage of staying within the balance of animal welfare concerns by minimizing injury, pain or discomfort, and largely staying within the boundaries of gaining or losing body weight. Furthermore, it allows for the preparation of small batches and therefore provides the ability to test or titrate new batches of drug. This diet gel-based oral drug delivery system may also be expanded to other drugs that are water soluble or water insoluble.

### Limitations of approach

The 93M liquification requires heating at 60 °C, which could inactivate heat-labile molecules during incorporation. Another limitation may be the difficulty of increasing dosages in 93M if the animals avoid the drug-infused diet due to palatability issues.

### Limitations of study

The real-time consumption behavior was not recorded. Differences between animals could change the outcomes depending on how quickly or slowly the animals eat the drug-infused 93M. Furthermore, the optimal daily consumption amount of 93M was defined for 8–12-week-old mice and may differ for other age groups. Another limitation is that we only investigated the effect of drug-infused 93M in the retina using one Cre line and a microglia reporter line. The effectiveness of the drugs could differ in other tissues or in combination with different Cre or reporter lines.

## Methods

### Animals

For all experiments, mice were single caged, and sex- and age-matched (between 8 and 12 weeks). Mice were maintained in a temperature and humidity‐controlled animal facility in individually ventilated cages on a 12‐h light–dark schedule. All animals had access to food according to the experimental design and to water ad libitum. Before each experiment mice were deprived of food for 16 h to increase appetite for a timely start of the experiment across all experimental animals and to reduce eventual reluctance of 93M consumption due to taste changes.

The study included three mouse strains: B6129SF1/J mice (RRID: IMSR_JAX:101043; The Jackson Laboratory), which were used for the evaluation of the optimal daily consumption rate of 93M. The *Cx3cr1*^*gfp/+*^ microglia reporter mice (B6.129P2(Cg)-*Cx3cr1*^*tm1Litt*^/J; RRID: IMSR_JAX:005582, The Jackson Laboratory) were used for the PLX5622 study. The Rlbp1-CreERT2*;Rosa*^*ai14*^ mice (Gt(ROSA)26Sor^tm14(CAG-tdTomato)Hze^ Tg(Rlbp1-cre/ERT2)1Eml/Eml; RRID: MGI:7708085; Levine Lab) were used for the tamoxifen study^21,22,48^. This study was approved by the Vanderbilt University Medical Center Institutional Animal Care and Use Committee, protocol number M1600235-02, and conformed to the Association for Research in Vision and Ophthalmology Statement for the Use of Animals in Ophthalmic and Vision Research.

### Experimental groups

Three experiments were designed. The first experiment evaluated the optimal daily consumption rate of 93M to ensure complete consumption while maintaining body weight by testing a low (6 g), an intermediate (8 g) and a high amount (10 g) of 93M provided daily in single-caged mice. The second tested the feasibility of PLX5622-infused 93M feeding as a dosage-controllable method to deplete microglia at a low (0.8 mg/g) and a high (2.0 mg/g) concentration. The third experiment tested whether tamoxifen can be administered through 93M feeding at a single concentration (312.5 μg/g) while controlling for the degrees of Cre recombination by adjusting the duration of the treatment (1 day versus 3-day treatment). Two comparison groups of oral gavage-treated animals (1-day versus 3-day treatment) were included to compare Cre recombination efficacy. For the 1-day treatment group, a tamoxifen concentration of 14.3 mg/ml was used to achieve a dosing of 143 µg/gbw. For the 3-day treatment group, a tamoxifen concentration of 9.1 mg/ml was used to achieve a daily dosing of 91 µg/gbw, yielding in a cumulative dosing of 273 µg/gbw. Vehicle-treated control groups (DMSO-93M) were included in the PLX5622- and tamoxifen-infused 93M experiments to assess a potential confounding effect of the drug vehicle and diet gel. The treatment regimen, fasting and data collection time points matched those of the drug-infused 93M groups.

### DietGel

Three different DietGel products provided by ClearH_2_O, Inc. were tested for drug−vehicle incorporation, which included DietGel Boost, DietGel 93M and DietGel GEM. DietGel 93M (93M) was selected for the study based on smoothness, liquefaction properties upon heating and emulsion properties of oil-based additives. The diet is a complete maintenance diet with enhanced flavor to promote consumption (https://clearh2o.com/products/dietgel%C2%AE-93m, accessed on 26 November 2024) and replaced the regular chow diet for the duration of the experiments.

### PLX5622 and tamoxifen incorporation into DietGel 93M

PLX5622 (C-1521, lot#21; Chemgood, LLC.) and tamoxifen (T5648, Sigma-Aldrich) were separately incorporated into 93M per cup. Each cup contained 75 g of 93M (measured in our laboratory).

PLX5622 was incorporated at two different concentrations to a final concentration of 0.8 mg/g and 2.0 mg/g. For the initial concentration, 60 mg of PLX5622 and 150 mg for the latter were each mixed into 1 ml of 100% DMSO (pharma grade, Heiltropfen Lab. LLP) using a 1.5 ml microcentrifuge tube. The tubes were protected from light and placed on a rocker for constant rocking for 1 h at room temperature, followed by a 20 min ultrasonic bath to achieve complete dissolvement.

Tamoxifen was used at a concentration of 312.5 µg/g. For one cup of 93M, 23.44 mg of tamoxifen was dissolved in 1 ml of corn oil (C8267, Sigma-Aldrich) on a rocker at room temperature overnight (light protected).

Each cup of 93M was heated in a water bath at 60 °C for 15 min to liquify 93M. Then 1 ml of the freshly prepared drug−vehicle solution was stirred in dropwise over 5 min using a P1000 pipet and a metal spatula, followed by 5 min of stirring to achieve a homogeneous mixture. The cups were sealed with Parafilm and placed on ice for 10 min before light-protected storage at 4 °C. The dug-infused 93M can be stored for at least up to 1 week at 4 °C.

To measure out the desired amount of DietGel for the experiment, a wooden single-use spatula was used to transfer it into a 5 cm glass Petri dish, which was then placed into the mouse cage. The dishes were thoroughly cleaned out daily with paper towels before new food was added.

### Anesthesia and SLO

The custom-built multimodal MURIN system, including a two-channel fluorescence SLO, was used for in vivo retinal fluorescence imaging of the left and right eye^49^. Mice were anesthetized using a mixture of isoflurane and oxygen set at a ratio of 2.5% and a flow rate of 1.5 l/min. Mydriasis was induced by applying one to two drops of 1% Tropicamide eye drops (VUMC pharmacy). The eyes were fitted with a custom zero-diopter contact lens to reduce optical aberrations at the air−cornea interface^49^. During imaging, 0.3% hydroxypropyl methylcellulose lubricant eye gel (GenTeal Tears; Alcon) was periodically applied to preserve corneal hydration. Animals were placed onto a custom water-heated imaging bed maintained at 38 °C with an integrated palate bar and nose cone to maintain anesthesia and reduce movement during the SLO imaging session. The green channel was used to image the GFP signal in the microglia-reporter mice B6.129P2(Cg)-*Cx3cr1*^*tm1Litt*^/J, and the red channel for the tdTomato expression in Müller glia of the Rlbp1-CreERT2*;Rosa*^*ai14*^ mice. Each SLO scan consisted of 200 acquired frames. Raw images were preprocessed as described elsewhere^49^.

### Quantification of longitudinal microglia counts and relative tdTomato expression in vivo in SLO scans

Preprocessed SLO images were loaded into Fiji (ImageJ 1.54f, National Institutes of Health) and 20−30 consecutive frames with minimal motion were selected for averaged intensity orthogonal projection. The projection image was converted to an 8-bit image followed by background subtraction with the rolling bar radius set at 20 pixels. The images were further processed for noise reduction and therefore sharpening using the unsharp mask function with a radius set at 6 pixels and a mask weight of 0.6 for the green channel (GFP) and 0.3 for the red channel (tdTomato), respectively^50,51^. A threshold was applied using the threshold function ‘Moments’. If needed, the threshold was manually adjusted to eliminate background noise. The region of interest (ROI) manager was used to outline the entire field of view, and the area fraction of positive pixels (% area) was automatically measured using the ‘Measure’ function after selecting ‘Limit to threshold’ and ‘Area fraction’ in the measurement settings. The area fraction is the relative tdTomato expression. Images of both eyes were included for quantification, with each eye considered a sample.

### Retinal whole-mount preparation and fluorescence microscopy

All mice were euthanized at the end of each experiment for endpoint histology using CO_2_ asphyxiation and secondary cervical dislocation. Both eyes were dissected using ophthalmic microscissors and rinsed in 1× PBS. The eyes were fixed in 4% PFA overnight at 4 °C. The right eye was temporarily stored in 1× PBS at 4 °C before processing for cryosectioning. The left eye was used for retinal whole-mount preparation and subsequent quantification. In brief, the cornea and lens were removed using ophthalmic microscissors. The sclera along with the choroid and retinal pigment epithelium were cut radially and peeled off to expose the retina and subsequently separated by cutting the optic nerve at its head. Next, four radial incisions were made into the retina before mounting on a glass slide with the ganglion cell layer facing up. The whole mounts were coverslipped using Fluoromount-G mounting medium (Invitrogen) and stored protected from light at 4 °C upon imaging.

The retinal whole mounts were scanned with wide-field epifluorescence using a ZEISS Axio Zoom.V16 with Apotome 3 structured illumination microscope at 40× digital magnification using a 1× objective. A 4 × 4 tile scan was performed to cover the entirety of the retina with a *z*-stack interval set at 9 µm. The scanning depth was individually adjusted to include all signal-positive layers. Apotome images were created using the software’s internal image processing function and tiles were fused with an overlap set at 20%.

### Endpoint quantification of microglia and relative tdTomato expression in retinal whole mounts

Apotome images were loaded into Fiji (ImageJ 1.54f, National Institutes of Health) for maximum intensity orthogonal projection. The projection image was converted to an 8-bit grayscale image. The background was subtracted with the rolling bar radius set at 50 pixels. A retinal ROI (rROI) outlining the retina without including the optical nerve head was created and saved by subtracting the optic nerve head area from the retinal area using the XOR function in the ROI manager. Next, separate image analysis protocols were used for images showing GFP-expressing microglia and tdTomato-expressing Müller glia.

Microglia reporter images were filtered using the morphological filtering operation ‘Top-hat’ with a radius set at 3 pixels to homogenize the background and enhance the morphological boundaries (contrast enhancement) for accurate automated cell counting^52–55^. A threshold was defined using the ‘Otsu’ settings and manually adjusted if needed. The ‘Analyze particles’ function was used for automated counting of individual cell bodies within the rROI. To do so, the size (micron^2^) was set to 10 – infinity, and ‘Count Masks’ and ‘Add to Manager’ were selected. The output files included ROIs of each counted cell. The new ROIs were overlayed onto the original image to confirm proper cell counting. If inaccurate, the counting was repeated with an adjusted threshold.

For the images showing tdTomato-expressing whole mounts, a threshold was defined using the function ‘Moments’ and, if necessary, manually adjusted to improve the background-to-signal ratio. To confirm proper thresholding, the function ‘Create selection’ was used to create an ROI outlining all positive pixels, which was then pasted onto the original image. The threshold was adjusted if the selection was deemed inaccurate. The rROI was applied to the threshold image before the ‘Measure’ function was used to calculate the area fraction (percentage area) of tdTomato positive pixels within the rROI. The area fraction is the relative tdTomato expression in the retinal whole mounts.

### Efficacy comparison in Cre recombination between 93M and OG delivered tamoxifen in retinal cryosections

Tamoxifen (T5648, Sigma-Aldrich) was dissolved in corn oil (C8267, Sigma-Aldrich) at a concentration of 14.3 mg/ml or 9.1 mg/ml, while 10 µl/gbw was delivered via oral gavage once (1-day treatment) or three times 24 h apart (3-day treatment), respectively. All mice were euthanized using CO_2_ asphyxiation and secondary cervical dislocation 14 days after the last tamoxifen gavage. Eyes were enucleated and rinsed in 1× PBS before overnight fixation in 4% PFA.

Next, the cornea and the lens were removed from the right eyes of the oral gavage-treated and the tamoxifen-infused DietGel-treated mice, followed by cryopreservation using a sucrose gradient of 5%, 10%, 20% and 30% in 1× PBS. The eye cups were embedded in OCT and oriented to avoid the low tdTomato expression area in the dorso-temporal retina during cutting. The embedded eye cups were stored at −80 °C. Cryosections were cut at a 12 µm thickness, and sections with the optic nerve present were serially mounted.

Immunohistology for Sox9 was used to identify nuclear Müller glia. In brief, sections were rinsed and permeabilized using 0.1% and 0.5% PBST (Tx-100). After blocking with 10% normal donkey serum in 0.1% PBST (Tx-100) for 1 h, rabbit anti-Sox9 (Ab5535, Chemicon, RRID: AB_2239761) was incubated overnight at a 1:500 concentration in 0.1% PBST (Tx-100) + 2% normal donkey serum. After washing 4× 10 min with 0.1% PBST (Tx-100), the sections were incubated with Alexa Fluor 488 donkey anti-rabbit IgG (H + L) (A-21206, Invitrogen, RRID: AB_2535792) at a 1:1,000 dilution for 2 h. After washing steps, the slides were cover-slipped using Fluoromount-G (0100-01, SouthernBiotech).

The retinal sections were scanned with wide-field epifluorescence using a ZEISS Axio Zoom.V16 with Apotome 3 structured illumination microscope at 200× digital magnification using a 2.3× objective, and a *z*-stack interval set at 1 µm. The scanning depth was individually adjusted to capture the entire thickness of the tissue. In total, five images were captured per eye after horizontal alignment with the inner nuclear layer. The areas were randomly selected across all sections in a nonadjacent manner. Apotome images were created using the software’s internal image processing function, followed by maximum intensity orthogonal projection. Each image served as an ROI for quantification.

The images were loaded into Fiji (ImageJ 1.54f, National Institutes of Health) for semi-automatic cell counting using the ‘Cell Counter’ plugin. All Sox9-positive and tdTomato Sox9 double-positive cells were counted. The efficacy was calculated as the average percentage of the double-positive over all Sox9-positive cells per image across the five images per eye.

### Statistics

The GraphPad Prism 10.4.1 software (GraphPad Software, Inc.) was used for data visualization and statistical analysis. Results are shown as mean ± s.e.m. A *P* value below 0.05 was considered significant.

### Reporting summary

Further information on research design is available in the Nature Portfolio Reporting Summary linked to this article.

## Online content

Any methods, additional references, Nature Portfolio reporting summaries, source data, extended data, supplementary information, acknowledgements, peer review information; details of author contributions and competing interests; and statements of data and code availability are available at 10.1038/s41684-025-01617-1.

## Supplementary information

Supplementary Information Supplementary Figs. 1−4

Reporting Summary

## Data Availability

The data generated during the current study are available from the corresponding authors upon request.
