# Supplementary material for: Diet gel-based oral drug delivery system for controlled dosing of small molecules for microglia depletion and inducible Cre recombination in mice
Source: Lab Anim (NY). 2025 Sep 26;54(10):278–85. doi: 10.1038/s41684-025-01617-1 (PMC12484080; doi:10.1038/s41684-025-01617-1)
Supplement: Supplementary file 1 — Supplementary Figs. 1−4 [file 41684_2025_1617_MOESM1_ESM.pdf]

lab animal

Supplementary information

https://doi.org/10.1038/s41684-025-01617-1

Diet gel-based oral drug delivery system for controlled dosing of small molecules for microglia depletion and inducible Cre recombination in mice

In the format provided by the authors and unedited

Supplementary Figure 1

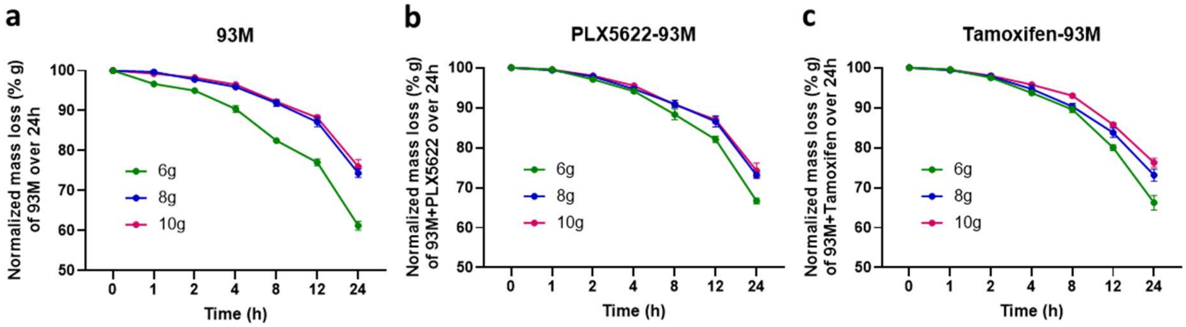

Supplementary Fig. 1| Temporal mass reduction of 93M with and without drug infusion due to evaporation. The different amounts of 93M (6 g, 8 g, 10 g) with and without drug infusions held in 5 cm glass petri dishes were placed individually into ventilated mouse cages. The weight was measured after 1, 2, 4, 8, 12 and 24 hours to establish mass reduction caused by evaporation (*n* = 4 per amount and drug group).

Supplementary Figure 2

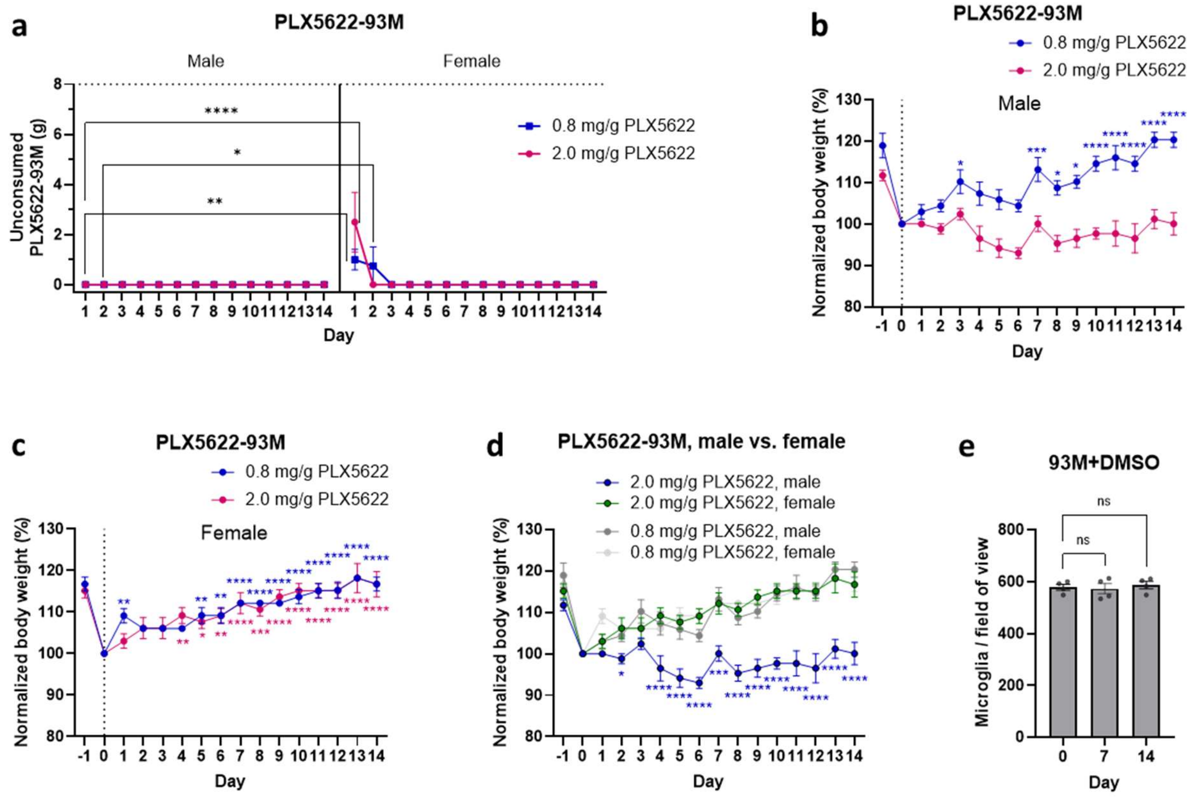

Supplementary Fig. 2| Supplementary data of the PLX5622-infused 93M experiment. a, The average mass of unconsumed PLX5622-infused 93M after each feeding cycle (24h) of the 0.8 mg/g and the 2.0 mg/g dosage group separated by sex (*n* = 4 per dosage group and sex). **b**, **c**, Temporal body weight measurements normalized and compared to the post-fasting body weight (day 0, dotted line) in males and females for both PLX5622-93M dosage groups (*n* = 4 per dosage group and sex). **d**, Aggregation of the data from (**b** and **c**). The shown significance levels are for the males (2.0 mg/g) in comparison to the female group (2.0 mg/g) at each time point. These males also significantly differ from the two other groups from day 4 onwards. **e**, Temporal quantification of GFP+ microglia in heterozygous B6.129P2(Cg)-*Cx3cr1* ^{tm1Litt}/J mice fed with 8 g/d of DMSO-infused 93M (vehicle-control) for 14 days using retinal *in vivo* SLO imaging (*n* = 4). Statistics: Two-way ANOVA with Tukey's multiple comparisons test for (**a**), two-way ANOVAs with Dunnett's multiple comparisons tests for (**b**,**c**), two-way ANOVA with Tukey's multiple comparisons test for (**d**), and one-way

ANOVA with Dunnett's multiple comparisons test for (**e**). Results are shown as Mean ± SEM. Significance levels: *$P < .05$, **$P < .01$, ***$P < .001$, ****$P < .0001$.

Supplementary Figure 3

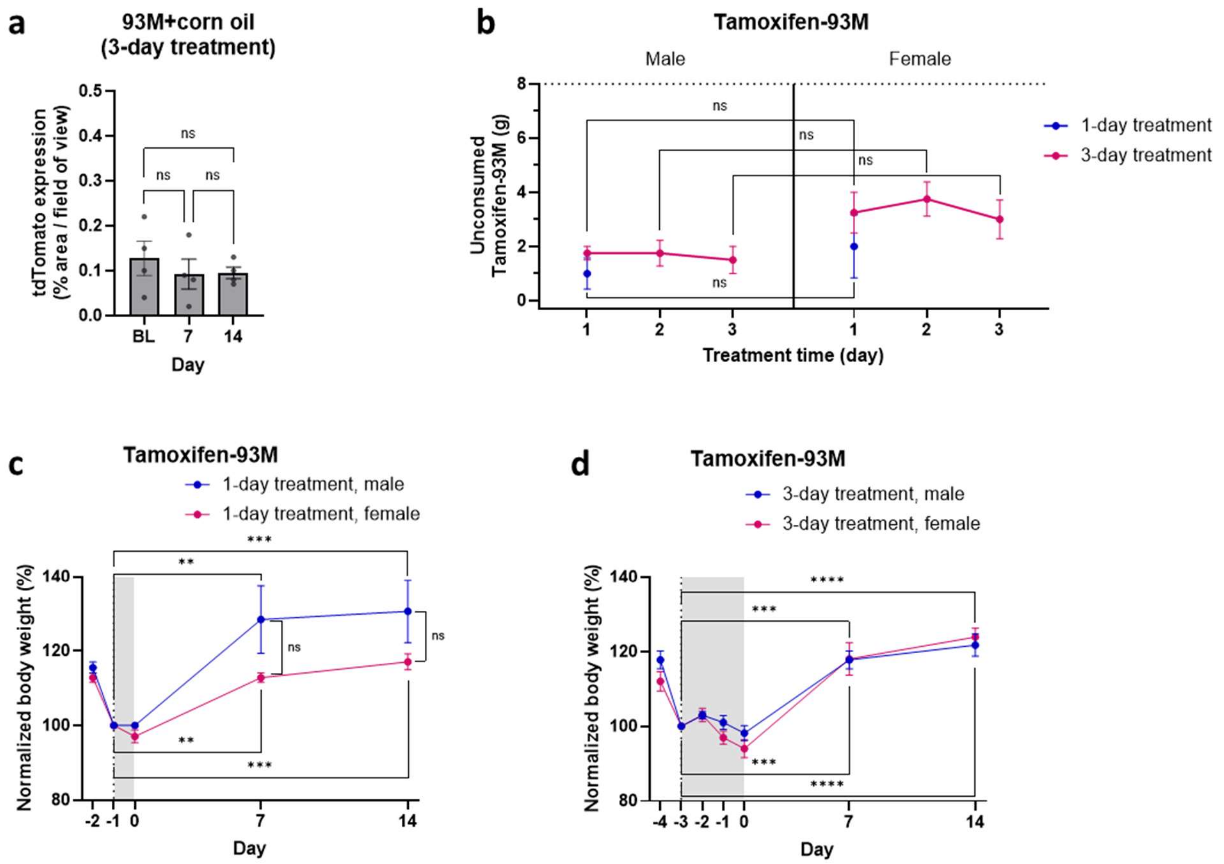

Supplementary Fig. 3| Supplementary data of the tamoxifen-infused 93M experiment. a, Temporal quantification of the tdTomato expression in heterozygous Rlbp1-CreERT2;*Rosa*^{ai14} mice fed with 8 g/d of corn oil-infused 93M (vehicle-control) for 14 days using retinal *in vivo* SLO imaging (*n* = 4). **b**, Averaged mass of unconsumed tamoxifen-infused 93M after each feeding cycle (24h) of the 1-day and 3-day treatment group separated by sex (*n* = 4 per treatment group and sex). **c**,**d**, Temporal body weight measurements normalized and compared to the post-fasting body weight (dotted line) following the 1-day and the 3-day treatment with tamoxifen-93M separated by sex (*n* = 4 per treatment group and sex). The gray shaded area indicates the treatment window of tamoxifen-93M exposure. Statistics: One-way ANOVA with Tukey's multiple comparisons test for (**a**), two-way ANOVA with Tukey's multiple comparisons test for (**b**), and two-way ANOVA with Sidak's multiple comparisons tests for (**c**,**d**). Results are shown as Mean ± SEM. Significance level: **$P < .01$, ***$P < .001$, ****$P < .0001$. Abbreviations: BL, baseline (pretreatment condition).

Supplementary Figure 4

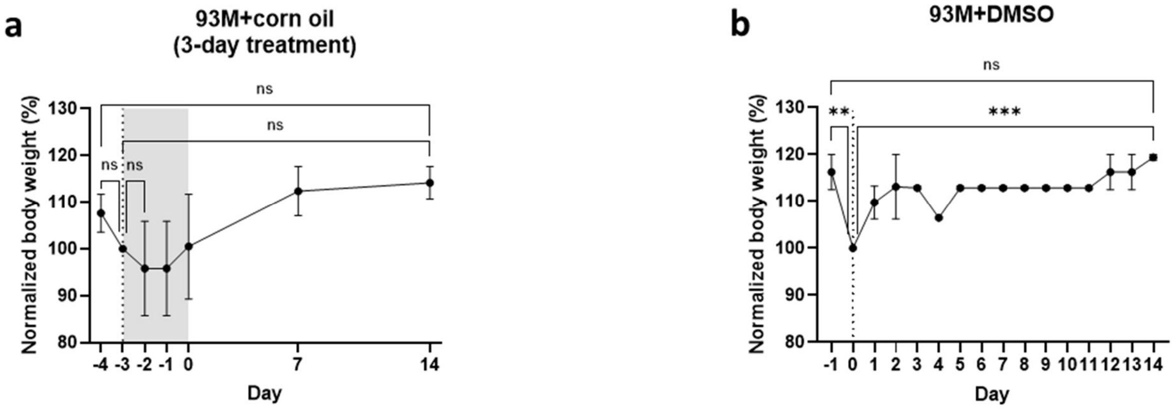

Supplementary Fig. 4| Supplementary data of the body weight measurements in the vehicle-infused 93M feeding paradigms (vehicle control). a,b, Temporal body weight measurements normalized and compared to post-fasting body weight (dotted line) in males and females for (**a**) corn oil-infused 93M and (**b**) DMSO-infused 93M. The Gray shaded area in (**a**) indicates the 3-day treatment window of tamoxifen-93M exposure. n = 2 mice per vehicle-infused 93M evaluation. Statistics: Two-way ANOVA with Tukey's multiple comparisons test for (**a**,**b**). Results are shown as Mean ± SEM. Significance levels: $^{**}P < .01$, $^{***}P < .001$.
