# Supplementary material for: Diet gel-based oral drug delivery system for controlled dosing of small molecules for microglia depletion and inducible Cre recombination in mice
Source: Lab Anim (NY). 2025 Sep 26;54(10):278–85. doi: 10.1038/s41684-025-01617-1 (PMC12484080; doi:10.1038/s41684-025-01617-1)
Supplement: Supplementary file 2 — Reporting Summary [file 41684_2025_1617_MOESM2_ESM.pdf]

nature portfolio

Corresponding author(s): Edward M. Levine
Joel Jovanovic

Last updated by author(s): Aug 17, 2025

Reporting Summary

Nature Portfolio wishes to improve the reproducibility of the work that we publish. This form provides structure for consistency and transparency in reporting. For further information on Nature Portfolio policies, see our Editorial Policies and the Editorial Policy Checklist.

Statistics

For all statistical analyses, confirm that the following items are present in the figure legend, table legend, main text, or Methods section.

n/a	Confirmed	
☐	☒	The exact sample size (*n*) for each experimental group/condition, given as a discrete number and unit of measurement
☐	☒	A statement on whether measurements were taken from distinct samples or whether the same sample was measured repeatedly
☐	☒	The statistical test(s) used AND whether they are one- or two-sided<br>*Only common tests should be described solely by name; describe more complex techniques in the Methods section.*
☐	☒	A description of all covariates tested
☐	☒	A description of any assumptions or corrections, such as tests of normality and adjustment for multiple comparisons
☐	☒	A full description of the statistical parameters including central tendency (e.g. means) or other basic estimates (e.g. regression coefficient) AND variation (e.g. standard deviation) or associated estimates of uncertainty (e.g. confidence intervals)
☒	☐	For null hypothesis testing, the test statistic (e.g. *F*, *t*, *r*) with confidence intervals, effect sizes, degrees of freedom and *P* value noted<br>*Give P values as exact values whenever suitable.*
☒	☐	For Bayesian analysis, information on the choice of priors and Markov chain Monte Carlo settings
☒	☐	For hierarchical and complex designs, identification of the appropriate level for tests and full reporting of outcomes
☒	☐	Estimates of effect sizes (e.g. Cohen's *d*, Pearson's *r*), indicating how they were calculated

Our web collection on statistics for biologists contains articles on many of the points above.

Software and code

Policy information about availability of computer code

Data collection	The body weight data and diet gel unconsumed amount were collected in Microsoft 365 Excel (Version 2411).<br><br>Scanning laser ophthalmoscopic (SLO) images of retinal microglia and tdTomato expression in vivo were collected using the MURIN system (SLO system) with a custom build software. The software and equipment is explained in: Rico-Jimenez, J. J. et al. MURIN: Multimodal Retinal Imaging and Navigated-laser delivery for dynamic and longitudinal tracking of photodamage in murine models. Frontiers in Ophthalmology 3, (2023).<br><br>Images of the retinal whole mounts and the retinal sections were captured with wide-field epifluorescence using a Zeiss Axio Zoom.V16 equipped with Apotome 3 structured illumination. Image volumes were post-processed using Apotome 3, followed by stitching with Zen 2.3 SP1 FP3 14.0 software. Final figures for publication were created in Affinity Designer 2, Version 2.6.3322.
Data analysis	Fiji (ImageJ 1.54f, National Institutes of Health) was used to quantify the microglia counts and the tdTomato area fraction in retinal whole mount projection images and in the SLO images, and to quantify the fraction of tdTom+Sox9+ over all Sox9+ MG cells in retinal section projection images, using a semi-automatic counting method described in the manuscript's method section.<br><br>GraphPad Prism 10.4.1 software (GraphPad Software, Inc., La Jolla, CA, USA) was used for statistical analysis and visualization of all data.

For manuscripts utilizing custom algorithms or software that are central to the research but not yet described in published literature, software must be made available to editors and reviewers. We strongly encourage code deposition in a community repository (e.g. GitHub). See the Nature Portfolio guidelines for submitting code & software for further information.

Data

Policy information about availability of data

All manuscripts must include a data availability statement. This statement should provide the following information, where applicable:

- Accession codes, unique identifiers, or web links for publicly available datasets
- A description of any restrictions on data availability
- For clinical datasets or third party data, please ensure that the statement adheres to our policy

The data generated during the current study are available from the corresponding authors upon request.

Research involving human participants, their data, or biological material

Policy information about studies with human participants or human data. See also policy information about sex, gender (identity/presentation), and sexual orientation and race, ethnicity and racism.

Reporting on sex and gender	n/a
Reporting on race, ethnicity, or other socially relevant groupings	n/a
Population characteristics	n/a
Recruitment	n/a
Ethics oversight	n/a

Note that full information on the approval of the study protocol must also be provided in the manuscript.

Field-specific reporting

Please select the one below that is the best fit for your research. If you are not sure, read the appropriate sections before making your selection.

☒ Life sciences ☐ Behavioural & social sciences ☐ Ecological, evolutionary & environmental sciences

For a reference copy of the document with all sections, see nature.com/documents/nr-reporting-summary-flat.pdf

Life sciences study design

All studies must disclose on these points even when the disclosure is negative.

Sample size	Pilot experiments using 93M-infused with either PLX5622 or tamoxifen showed a strong drug-effect. Based on this knowledge, sample sizes were set at 8 mice (4 male, 4 females) per treatment group. There were 4 treatment groups overall with two PLX5622-doses and two treatment-time dependent tamoxifen treated groups. For the tamoxifen efficacy comparison between the oral gavage and the 93M apporach, 3 animals with similar dosing in the dose-response curve were identified per treatment regimen (1 day vs 3 day), and the oral gavage comparison mice were matched in numbers and sex.
Data exclusions	No data was excluded. For the 93M consumption rate determination experiment (Figure 1), one mouse in the male group fed with 6 g/d was taken out of the study on day 12 due to a body weight loss over 30%. The collected data upon that date was included for data analysis.
Replication	The experiments for the PLX5622 and Tamoxifen treatment were split into groups, divided by sex, and sequentially executed. Thus, replication occurred at the level of sex-separation. Furthermore, longitudinal data collection in vivo of the same mouse allows for each mouse to serve as an individual replication. In addition, pilot studies were done for each drug (using different Lots of PLX5622, or concentrations of Tamoxifen) to establish the experimental conditions. These pilot studies introduce a third level of replication.
Randomization	Individual animals were randomly assigned to the treatment groups within the confines of genotypes, sex and the number of animals per treatment group.
Blinding	Blinding was introduced for the quantification of images, where the file names did not include specific identifiers of conditions. Conditions were unblinded when quantification was complete.

Reporting for specific materials, systems and methods

We require information from authors about some types of materials, experimental systems and methods used in many studies. Here, indicate whether each material, system or method listed is relevant to your study. If you are not sure if a list item applies to your research, read the appropriate section before selecting a response.

Materials & experimental systems

n/a	Involved in the study
☐	☒ Antibodies
☒	☐ Eukaryotic cell lines
☒	☐ Palaeontology and archaeology
☐	☒ Animals and other organisms
☒	☐ Clinical data
☒	☐ Dual use research of concern
☒	☐ Plants

Methods

n/a	Involved in the study
☒	☐ ChIP-seq
☒	☐ Flow cytometry
☒	☐ MRI-based neuroimaging

Antibodies

Antibodies used	- Rabbit α-Sox9, Chemicon, Cat# Ab5535; RRID: AB_2239761<br>- Alexa Fluor 488 Donkey Anti-Rabbit IgG (H+L), Invitrogen, Cat# A21206; RRID: AB_2535792
Validation	Both antibodies are established in the lab and have been carefully tested in relation with other projects, by applying the primary and no secondary antibody, and the secondary only without primary, all in retinal cryosections. The Muller glia-specific endogenous tdTomato reporter served as an additional marker to correclty identify Muller glia cells by the Sox9 and Alexa Fluor 488 antibodies used for this manuscript. The antibodies have also been used for a previous publication (PMID: 39628577).

Animals and other research organisms

Policy information about studies involving animals; ARRIVE guidelines recommended for reporting animal research, and Sex and Gender in Research

Laboratory animals	We focused on three mouse strains at the age of 8-12 weeks. The following three strains were induced:<br>1. B6129SF1/J mice (RRID:IMSR_JAX:101043; The Jackson Laboratory, ME, USA) were used for the evaluation of the optimal daily consumption rate of 93M.<br>2. Cx3cr1gfp/+ microglia reporter mice (B6.129P2(Cg)-Cx3cr1tm1Litt/J; RRID:IMSR_JAX:005582, The Jackson Laboratory, ME, USA) were used for the PLX5622 study.<br>3. Rlbp1-CreERT2;Rosaai14 mice (Gt(ROSA)26Sortm14(CAG-tdTomato)Hze Tg(Rlbp1-cre/ERT2)1Eml/Eml; RRID:MGI:7708085; Levine Lab, VUMC, TN, USA) were used for the Tamoxifen study.
Wild animals	The study did not involve wild animals.
Reporting on sex	For this study, sex was considered as a variable. Mice were sex and age matched across all experiments. Effects of PLX5622 and Tamoxifen treatment were evaluated separately for male and females using the SLO in vivo measurements. Since no sex-specific effect was observed by SLO, histological confirmation of the SLO results was calculated across both sexes within each treatment group.
Field-collected samples	This study did not include field-collected samples.
Ethics oversight	This study was approved by the Vanderbilt University Medical Center Institutional Animal Care and Use Committee and conformed to the Association for Research in Vision and Ophthalmology Statement for the Use of Animals in Ophthalmic and Vision Research.

Note that full information on the approval of the study protocol must also be provided in the manuscript.

Plants

Seed stocks	n/a
Novel plant genotypes	n/a
Authentication	n/a
